# Appointments by Health Commissioner Fronczak

**Published:** 1910-05

**Authors:** 


### Appointments by Health Commissioner Fronczak

Dr. Walter S. Goodale of No. 90 North Pearl street, who has been temporary superintendent of the municipal hospital at Broadway and Spring street for about a year, was permanently appointed to that position March 31, 1910, by Health Commissioner Fronczak. Dr. Goodale was at the head of the civil-service list prepared after the recent examination. The salary is $1,200 a year.

Other appointments announced also by the Health Commissioner were:

Dr. Jesse N. Roe, No. 456 Broadway, resident physician at the hospital, salary $900.

Marjorie G. Carney, matron of the hospital, salary $900.

Dr. B. Ross Nairn, No. 295 Baynes street, medical school inspector in place of Dr. Arthur C. Schaefer, lately appointed assistant health commissioner; salary of inspector, $1,000 for ten months' service.

Dr. J. H. Wild, No. 528 Elm street, homeopathic physician for the East Side, salary $200 a year and allowances for prescriptions.

---

New York is likely to experience a quiet Fourth of July, unless Mayor Gaynor's orders are disobeyed or are rescinded. His expressed determination to prohibit the retailing of fireworks between June 10, and July 10, will put the small boy out of commission as a creator of noise and smoke and lockjaw on the anniversary of the nation's independence. It should be possible to devise some plan for observing the day that would be satisfactory to young Americans, without offending the proprieties of the occasion, without disturbing the sick, and without endangering life, limb, or property. We sincerely hope Buffalo will take action in the premises.

---

THE cold storage time limit is nine months, according to Dr. H. W. Wiley, Chief of the Bureau of Chemistry, Department of Agriculture, in a report recently presented to Senator Lodge, chairman of the committee charged with investigating the causes of the high cost of living.

"Whenever the storage of a product is continued long enough to interfere with the output of the next season's crop," says Dr. Wiley, "it has been carried to a point beyond the limits of reasonable economy and practical hygiene." He says that the storage of strictly seasonable products should be prohibited by law, and that even canned goods, which are a benefit to the people, should not be carried over longer than the ending of one season, and the beginning of the next.

---

OYSTERS can no longer be fed—or, as the trade term goes, floated in brackish water"—before they are offered for sale. The Department of Agriculture has ordered that the practice must stop at once. The order affects the entire oyster trade of the United States, and is of immense importance to dealers.

The department recently gave a hearing to the oystermen, who maintained that the quality of the oyster is improved by the floating process. The department, however, holds that to "float" an oyster after it is taken from its bed gives means for the oyster to take on contamination from the water and offers risk of typhoid. Oystermen from the New Jersey beds came in an organisation two hundred strong to Washington a few weeks ago to protest against the order, holding its enforcement would ruin them.

---

DR. J. H. PIERCE, a prominent veterinary surgeon of Wellsville, died April 15, 1910, in New York of hydrophobia. Dr. Pierce was bitten by a dog owned by Mrs. Hathaway of Wellsville several weeks ago.

The usual precautions were taken. The dog was killed and its brain sent to Cornell University for examination. The experts there pronounced it a case of rabies, and further precautions were taken to prevent the development of the disease in Dr. Pierce.

Symptoms of hydrophobia developed early in the week of his death, whereupon he was sent to the Pasteur Institute in New York, in the hope of saving his life.

Dr. James S. Cumming, director of the Pasteur Institute of the University of Michigan, announced April 1, 1910, the discovery of a new method for the treatment of hydrophobia. The new treatment, Dr. Cumming says, eliminates many of the dangers attending the former methods and shortens the time of treatment by one week.

Dr. Cumming uses a virus prepared from spinal tissues of a rabid animal. This is injected into the patient. The virus is said to have been used in several cases recently with marked success. It is a pity this method was not tried in the case of Dr. Pierce.

